# Abnormal Homeostasis in the Redox State and Related Signaling Pathways, in Irritable Bowel Syndrome

**DOI:** 10.1111/nmo.70097

**Published:** 2025-06-22

**Authors:** A. S. Morales‐Guzmán, A. Alarcón‐Aguilar, A. Luna‐López, A. D. Santana‐Vargas, M. Motola‐Kuba, R. Librado‐Osorio, J. A. García‐Álvarez, M. J. Schmulson

**Affiliations:** ^1^ Laboratory of Liver, Pancreas and Motility (HIPAM), Department of Experimental Medicine Dr. Ruy Pérez Tamayo Faculty of Medicine, Universidad Nacional Autónoma de México (UNAM) Mexico City Mexico; ^2^ Postgraduate School in Experimental Biology, Directorate of Biological and Health Sciences (DCBS) Universidad Autónoma Metropolitana (UAM)‐Unidad Iztapalapa Mexico City Mexico; ^3^ Laboratory of Bioenergetics and Cellular Aging, Department of Health Sciences Universidad Autónoma Metropolitana (UAM)‐Unidad Iztapalapa Mexico City Mexico; ^4^ Department of Basic Research Instituto Nacional de Geriatría Mexico City Mexico; ^5^ Department of Clinical Research Hospital General de México Dr. Eduardo Liceaga Mexico City Mexico; ^6^ Gastroenterology Service Hospital General de México Dr. Eduardo Liceaga Mexico City Mexico; ^7^ Department of Cell Biology Faculty of Science, Universidad Nacional Autónoma de México (UNAM) Mexico City Mexico

**Keywords:** gp91^phox^, irritable bowel syndrome, NF‐κB, Nrf2, oxidative stress

## Abstract

**Background:**

IBS is multifactorial; however, elucidating its underlying mechanisms is crucial for advancing in its diagnosis and management.

**Aims:**

Evaluate molecular processes related to oxidative stress (OS) and inflammation in IBS and its subtypes.

**Methods:**

Thirty Rome III‐IBS outpatients and 30 controls were studied for OS biomarkers, including malondialdehyde (MDA), protein carbonyls (PC), reduced glutathione (GSH), and oxidized glutathione (GSSG). Also, serum interleukins (IL‐10, IL‐4, TNF‐α, IL‐6), and the nuclear factor kappa‐light‐chain‐enhancer of activated B cells (NF‐κB), nuclear factor erythroid 2‐related factor 2 (Nrf2), and nicotinamide‐adenine‐dinucleotide phosphate (NADPH) catalytic subunit gp91^phox^.

**Results:**

In IBS vs. controls there were higher MDA: 4.44 ± 1.76 vs. 2.42 ± 0.5 nmol/mg/protein (*p* < 0.01); GSSG: 57.17 ± 17.49 vs. 42.73 ± 14.26 μM (*p* < 0.01); and lower GSH: 26.17 ± 12.36 vs. 38.47 ± 16.71 μM (*p* < 0.01). Also, an imbalance in pro‐ and anti‐inflammatory interleukins (*p* < 0.01); and higher NF‐κB: 5.33 ± 3.39 vs. 3.08 ± 1.19 (*p* = 0.01); gp91^phox^: 4.28 ± 1.81 vs. 3.29 ± 1.03 (*p* < 0.05); and lower Nrf2: 3.87 ± 2.9 vs. 7.56 ± 2.59 (*p* < 0.05). Additionally, there were no significant differences between the IBS subtypes, nor according to severity. Finally, in IBS‐C, MDA correlated with IL‐4, TNF‐α with IL‐10; and in IBS‐D, GSH correlated with IL‐4 and no differences in transcription factors.

**Conclusions:**

The data demonstrate an alteration in the homeostasis of the cellular redox state in IBS. Also, in IBS‐D, the antioxidant effect counteracts the low‐grade inflammation, whereas in IBS‐C, it is mainly driven by interleukins.

## Graphical abstract

There is higher oxidative stress and higher expression of transcription factor NF‐κB and the gp91 subunit of NADPH oxidase in IBS, with lower antioxidant regulator Nrf2. There are no differences in the IBS subtypes; however, in IBS‐C, malondialdehyde correlated with IL‐4, and TNF‐α with IL‐10, suggesting an interleukin‐mediated anti low‐grade inflammation mechanism, and in IBS‐D, GSH correlated with IL‐4, suggesting an antioxidant mechanism.

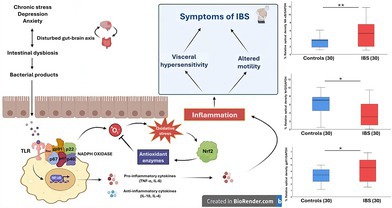

**Summary**

- Proposed molecular mechanism in IBS.
  ○ IBS can develop due to stress, and this can cause an alteration in the gut–brain axis, resulting in intestinal dysbiosis and allowing the translocation of antigens.
  ○ The antigens activate the immune system and follow a signaling pathway that results in the activation of NADPH oxidase that induces activation of transcription factor NF‐κB. It controls the expression of various pro‐inflammatory mediators and genes involved in stress signaling pathways.
  ○ On the other hand, when translocated antigens are in the deeper layers of the gut, NADPH oxidase is activated and produces ROS.
  ○ However, dysregulation of NADPH oxidase activity can create an oxidative environment and contribute to intestinal inflammation with subsequent symptoms of IBS.
  ○ The transcription factor Nrf2 is activated in oxidative stress conditions to regulate the expression of genes encoding antioxidant enzymes, inhibit the expression of pro‐inflammatory genes, and attenuate inflammation.
  ○ In our study, patients with IBS show greater expression of NF‐κB: 5.33 ± 3.39 vs. 3.08 ± 1.19 (*p* < 0.01), and gp91^phox^ subunit:….. 4.28 ± 1.81 vs. 3.29 ± 1.03 (*p* < 0.05), and lower expression of Nrf2: 3.87 ± 2.99 vs. 7.56 ± 2.59 (*p* < 0.05).

## 1 Background

Irritable bowel syndrome (IBS) is a highly prevalent disorder of gut–brain interaction (DGBI), previously called functional gastrointestinal disorders (FGID) [1], characterized by abdominal pain and abnormal bowel habits [2]. The Rome Foundation Global Epidemiological Study carried out in the general population found a global IBS prevalence of 4.1% (95% confidence interval [95% CI]: 3.9–4.2), and 4.0% (IC 95%: 3.2–4.9) in Mexico. IBS diagnosis is based on symptom criteria, the Rome criteria; and based on the predominant bowel habit, it is classified into IBS with diarrhea (IBS‐D); IBS with constipation (IBS‐C); mixed IBS (IBS‐M); and unclassified IBS (IBS‐U) [3].

IBS pathophysiology is not completely understood. It involves a complex interaction between several factors with disturbances in three main connectomes with bidirectional communications among them, including the gut, the gut microbiome, and the brain, which are now integrated into what is called the microbiome–gut–brain axis [4]. The abnormalities within each of these nodes include gut dysbiosis; immune function, low‐grade mucosal inflammation, and increased epithelial permeability in the gut; visceral hypersensitivity; and central nervous system (CNS) dysregulation. Between the CNS and the gut, the autonomic nervous system mediates alterations in gut motility, secretion, visceral hypersensitivity, and the central perception of gut sensations [5].

The altered immune system changes in the gut have gained interest in recent years [6]. The normal gastrointestinal immune system is under strict regulation and balance of cytokines and other inflammatory and anti‐inflammatory mediators. This balance is essential to maintain normal intestinal function [7] (e.g., some interleukins have pro‐ while others have anti‐inflammatory effects). For example, the inflammatory response is mediated through tumor necrosis factor‐alpha (TNF‐α) and interleukin‐6 (IL‐6), whereas the anti‐inflammatory response is mediated through interleukin‐10 (IL‐10) and interleukin‐4 (IL‐4), among others [8]. The imbalance between pro‐inflammatory and anti‐inflammatory interleukins may contribute to inflammation and visceral sensitization, leading to the characteristic symptoms of IBS [9]. In addition, several interleukins can stimulate the nuclear factor kappa‐light‐chain‐enhancer of activated B cells (NF‐κB) [10]. The activation of NF‐κB plays a central role in the inflammatory and immune response [11, 12]. This creates a positive feedback loop, as the produced interleukins can, in turn, further activate NF‐κB signaling [13, 14, 15]. Chronic inflammation can sustain oxidative stress (OS) by perpetuating the production of reactive oxygen species (ROS) and reactive nitrogen species (RNS) through the activation of immune cells and the recruitment of additional inflammatory mediators [16, 17]. OS happens when there is an imbalance between the production of ROS and the production of antioxidant enzymes, so the cells lose the redox state (balance between ROS and antioxidant enzymes). The high levels of ROS result in damage to biomolecules such as lipid peroxidation, which is a common consequence of OS, leading to the formation of malondialdehyde (MDA). Also, OS can cause the oxidation of amino acid side chains in proteins, forming carbonyl groups. However, enzymatic antioxidants like glutathione (GSH), vitamin C, vitamin E, and various polyphenols can scavenge ROS and protect cells from oxidative damage [18]. Measurement of the levels of these antioxidants can provide insights into the antioxidant defense system's status and its ability to counteract OS [19].

On the other hand, the nuclear factor erythroid 2‐related factor 2 (Nrf2) is a transcription factor that plays a crucial role in protecting cells from oxidative damage and maintaining cellular homeostasis [20]. When cells are exposed to OS, Nrf2 is activated and translocated into the nucleus, which binds to antioxidant response elements (ARE) in the promoter regions of target genes [21, 22]. Therefore, the interaction between Nrf2 and NF‐κB is essential to maintain the physiological homeostasis of the redox state and regulate the response to stress and inflammation [23].

Finally, nicotinamide adenine dinucleotide phosphate (NADPH) oxidase is a transmembrane enzyme found in various cells of the immune system and is one of the main sources of ROS generation, such as the superoxide anion (O_2_^−^) by transferring electrons from NADPH to molecular oxygen (O_2_) [24]. NADPH oxidase comprises six subunits; among them, subunit gp91^phox^ is the catalytic unit that transfers electrons from cytosolic NADPH to (O_2_), producing (O_2_^−^) [25]. This pathway is an important microbial defense mechanism as ROS are toxic to microorganisms, thus contributing to the immune response against infections [26]. Therefore, the proper balance in NADPH oxidase activity is essential for the maintenance of redox homeostasis, and its poor regulation can generate OS; consequently, influencing the severity of low‐grade inflammation and the bacterial composition in the colon [27, 28].

OS may play a central role in the pathophysiology of IBS by disrupting the balance between free radicals and antioxidant mechanisms. It is plausible that this imbalance leads to the activation of several intracellular pathways that perpetuate inflammation and cause damage to the tight junctions [29]. OS in IBS is rather a perpetuating element within a vicious cycle in which factors, such as dysbiosis, inflammation, and psychological stress contribute to OS, which in turn amplifies these alterations [30]. Therefore, this study aimed at evaluating the molecular processes related to OS and inflammation in IBS, as well as within the different IBS subtypes.

## 2 Material and Methods

### 2.1 Study Subjects

The study included 30 IBS outpatients of the Gastroenterology Clinic of the *Hospital General de México Dr. Eduardo Liceaga (HGM)*, in Mexico City. The HGM is a general hospital, and it is the largest one of the health system of Mexico's Secretary of Health. IBS‐negative controls were sex and age matched and were recruited from the accompanying relatives in the waiting room of the same HGM. Exclusion criteria were recent systemic or focal infections (less than 1 month), chronic degenerative disease (e.g., diabetes, cancer, celiac disease, inflammatory bowel disease, cancer, autoimmune diseases), history of major gastrointestinal surgeries except for appendectomies and/or cholecystectomies, and use of anti‐inflammatory medications, antioxidants, and/or food supplements.

IBS was diagnosed according to the Rome III criteria, and the IBS subtype was further determined. For this purpose, all participants answered the Rome III Adult Questionnaire for FGID, validated in Spanish‐Mexico. In addition, subjects fulfilling Rome III criteria for IBS answered the IBS‐Symptom Severity Scale (IBS‐SSS) in Spanish‐Mexico. Severity was classified as mild: 75–175, moderate: 175–300, and severe: > 300.

### 2.2 Biochemical Analysis

Peripheral venous blood samples were collected from all participants. Whole blood samples were collected in vacutainer tubes and centrifuged; serum, plasma, and blood were frozen at −80°C until processing.

### 2.3 Measurement of Serum MDA Levels

Lipid peroxidation was assessed by the thiobarbituric acid‐reactive substances (TBARS) formation, according to a previous report [31], based on the reaction of MDA with thiobarbituric acid (TBA) at 95°C. In the TBA test reaction, MDA and TBA form a pink pigment with an absorption maximum of 532 nm.

### 2.4 Measurement of GSH, GSSG, and GSH/GSSG

Measurement of plasma redox state (GSH/GSSG ratio). In the samples, GSH, GSSG, and GSH/GSSG concentrations were determined with the 38185 quantification kit for oxidized and reduced glutathione (Sigma‐Aldrich). The kit is based on the Ellman method. The solution derived from a colorimetric reaction is determined by measuring the absorbance at 415 nm.

### 2.5 Measurement of Serum Protein Carbonyls (PC) Levels

In the samples, PC concentrations were determined to assess protein oxidative damage using the 2,4‐dinitrophenylhydrazine (DNPH) alkaline method reaction [32] to form 2,4‐dinitrophenylhydrazone. The dinitrophenyl group can be detected and quantified spectrophotometrically because it exhibits a characteristic absorption spectrum with a maximum absorbance of 365–375 nm.

### 2.6 Protein Content Determination

Total protein concentration was determined using the bicinchoninic acid (BCA), Pierce BCA Protein Assay Kit (Thermo‐Scientific), according to the manufacturer's instructions.

### 2.7 Interleukins Levels

The serum levels of the pro‐inflammatory interleukins TNF‐α, IL‐6, and the anti‐inflammatory IL‐10 and IL‐4 were measured by the commercially available enzyme‐linked immunosorbent assay (ELISA MAX SET [BioLegend]). The assay was performed following the manufacturer's instructions. The data were obtained from a spectrophotometer instrument, and the concentration of the analytes was measured following the manufacturer's recommendations.

### 2.8 Western Blot Analysis

The blood samples were lysed in T‐Per buffer (tissue Protein Extraction Reagent). T‐Per buffer was prepared (100 μL of 1 M DTT (dithiothreitol), 100 μL of 0.1 M PSMF (phenylmethylsulphonyl fluoride), and a mini complete tablet (Roche), suspended in 10 mL of T‐Per buffer (sigma)). After the determination of protein concentration, proteins (100 μg) from each fraction protein were separated on 12% SDS‐PAGE and transferred to polyvinylidene fluoride (PVDF) membranes (membrane of PVDF Immobilon‐FL). The membranes were incubated with primary antibodies (1:1000 for NF‐kB/p65 [SC‐8008], 1:5000 for Nrf2 [MA5‐33211], 1:1000 for gp91 [SC‐5827], and 1:1000 for GAPDH [SC‐32233]) overnight at 4°C. Subsequently, the membranes were incubated with the appropriate secondary antibody dilution (1:1000; Santa Cruz Biotechnology, Santa Cruz, CA, USA) and detected by the (Oddysey CLx). The intensity of each band was quantified using NIH ImageJ Software (version 1.54).

### 2.9 Statistical Analysis

Data were compared using the Student's *t*‐test or the *U* Mann–Whitney test and analysis of variance with Turkey post hoc was used when appropriate. A *p* < 0.05 was considered significant. SPSS Statistics 22.0 software was used for data management and statistical analyses.

The protocol was approved by the Investigation and Ethic Commissions of the Faculty of Medicine of the *Universidad Nacional Autónoma de México* (UNAM), and the *Hospital General de México Dr. Eduardo Liceaga*. All participants were informed about the study protocol, and written consent was obtained from each of them.

## 3 Results

### 3.1 IBS Patients and Controls

The 30 Rome III‐IBS patients included 27 women and 3 men; age range: 18–65 years; and the 30 IBS‐negative controls were 27 females and 3 males; age range: 18–62 years. Also, there were no differences in age according to sex between the groups (Table 1). According to the IBS subtype, almost half were IBS‐U, followed by 11 patients with IBS‐C, four with IBS‐D, and only one with IBS‐M. Thus, the IBS‐M was excluded from the analysis between IBS subtypes for statistical purposes (Table 1). The severity of IBS was mild in 4 (13%) patients, IBS‐SSS score of 146.50 ± 17.00; moderate in 12 (40%), 256.45 ± 30.20; and severe in 14 (47%), 370.33 ± 53.56 (Table 1).

**TABLE 1:** General demographic characteristics of the study groups.

Group	Age (mean ± SD)	Women, *n* (%)	Men, *n* (%)	IBS‐subtype	Age	Women, *n* (%)	Men, *n* (%)
IBS	42.0 ± 14.0	27 (90)	3 (10)	IBS‐U	42.0 ± 13.0	13 (93)	1 (7)
				IBS‐C	41.4 ± 16.5	10 (91)	1 (9)
				IBS‐D	50.5 ± 15.3	3 (75)	1 (25)
				IBS‐M	19	1	—
Controls	42.0 ± 15.0	27 (90)	3 (10)				
*p*	0.93	0.89	0.85				

*Note:* Data are shown as mean ± SD.

Abbreviations: IBS, irritable bowel syndrome; IBS‐C, constipation predominance; IBS‐D, diarrhea predominance; IBS‐M, mixed predominance; IBS‐U, unclassified predominance; SD, standard deviation.

### 3.2 Oxidative Stress (OS)

**FIGURE 1:**
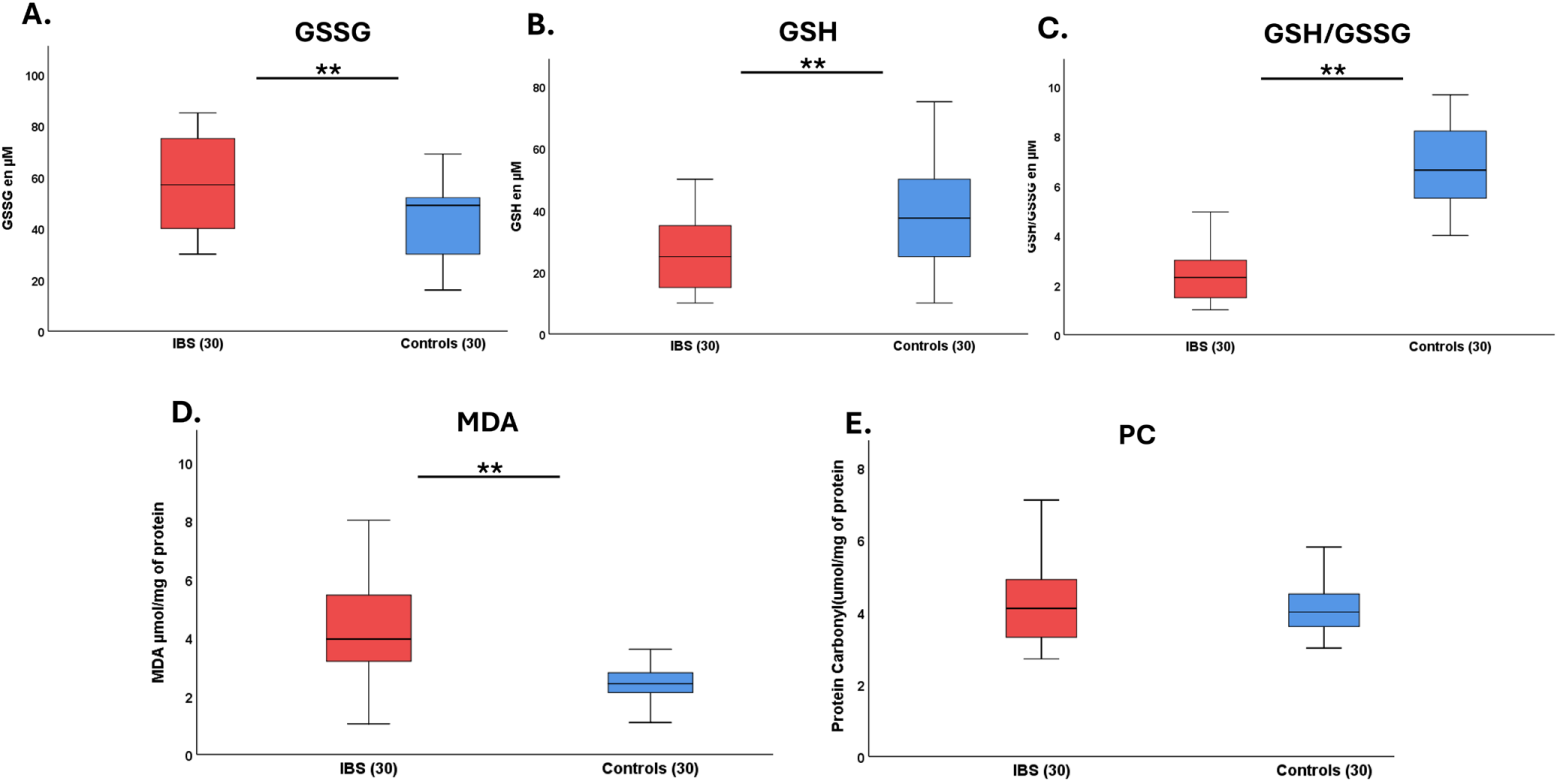
Mean levels of oxidative stress in patients with IBS and controls. Graphs show levels of GSSG μM (A), GSH μM (B), GSH/GSSG ratio (C), MDA μmol/mg (D), and PC μmol/mg (E). Data are shown as mean ± SD, *n* = 30. **p < 0.01.

The IBS patients showed higher concentrations of GSSG than controls: 57.17 ± 17.49 vs. 42.73 ± 14.26 μM (*p* < 0.01) (Figure 1A); higher MDA: 4.44 ± 1.76 vs. 2.42 ± 0.5 nmol/mg/protein (*p* < 0.01) (Figure 1D); lower concentrations of GSH: 26.17 ± 12.36 vs. 38.04 ± 16.71 μM (*p* < 0.01) (Figure 1B); and a lower GSH/GSSG ratio: 2.42 ± 1.09 vs. 6.76 ± 1.68 (*p* < 0.01) (Figure 1C). No statistically significant differences were observed in PC levels between patients and controls (*p* > 0.05) (Figure 1E). Furthermore, there were no significant differences in the OS biomarkers between the IBS subtypes (Table 2). Also, there were no significant differences in the OS markers according to IBS severity (Table S1).

**TABLE 2:** Mean levels of oxidative stress in different bowel habit subtypes of IBS.

Subtypes	GSSG (μM)	GSH (μM)	GSH/GSSH ratio	MDA (μmol/mg/protein)	PC (μmol/mg/protein)
IBS‐U (14)	50.23 ± 10.02	20.07 ± 10.15	2.37 ± 1.20	5.01 ± 3.11	4.4 ± 1.1
IBS‐C (11)	50.27 ± 10.35	20.31 ± 10.02	2.28 ± 0.99	6.15 ± 5.13	4.6 ± 0.6
IBS‐D (4)	49.56 ± 20.05	20.14 ± 10.05	1.89 ± 1.02	2.23 ± 1.04	3.7 ± 1.3
*p*	0.91	0.98	0.95	0.25	0.83

*Note:* Data are shown as mean ± SD, *n* = 29.

Abbreviations: IBS‐C, constipation predominance; IBS‐D, diarrhea predominance; IBS‐U, unclassified predominance.

### 3.3 Serum Interleukins

**FIGURE 2:**
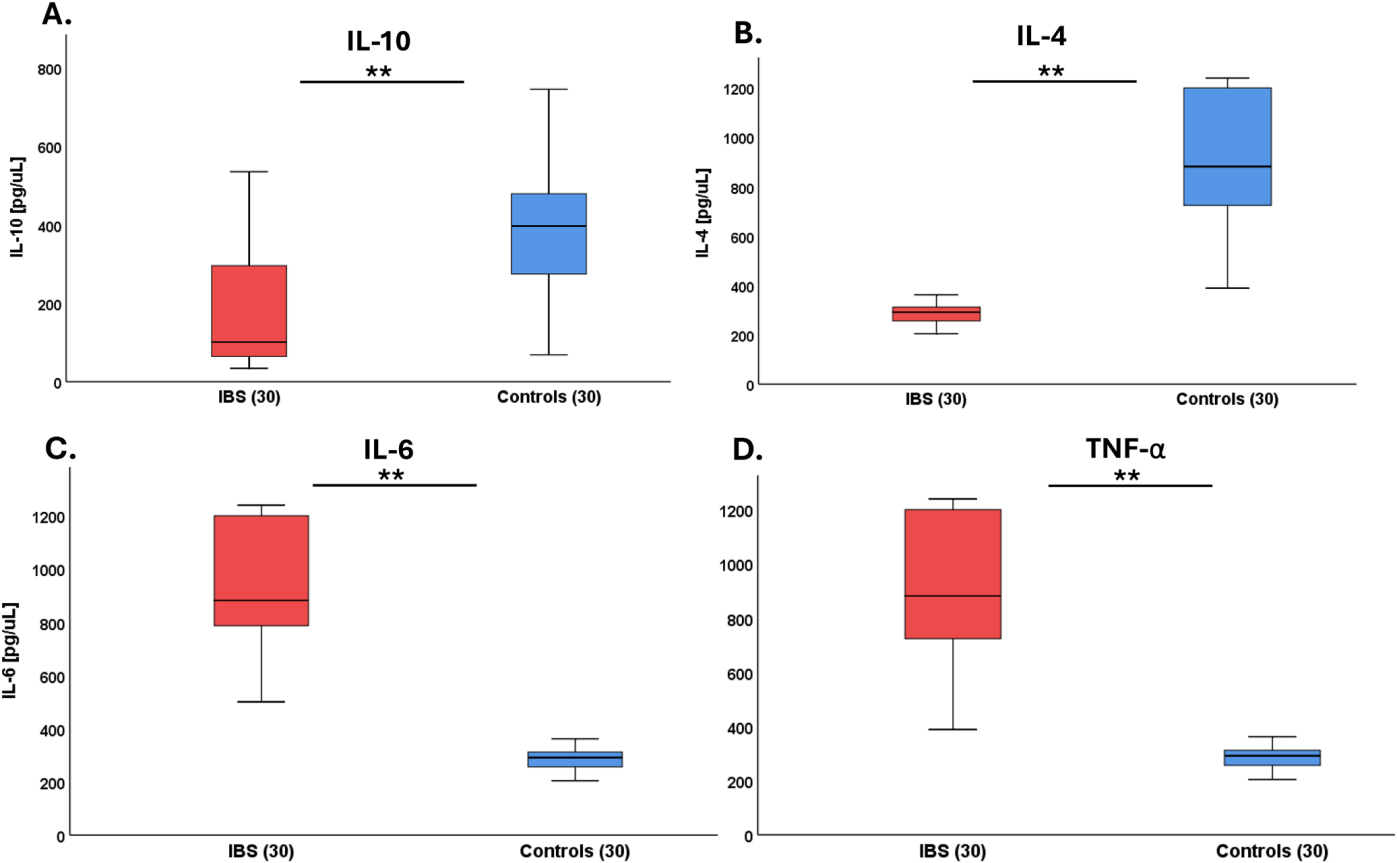
Mean serum levels of inflammatory interleukins and anti‐inflammatory interleukins in patients with IBS and controls. Graphs show the interleukin levels of IL‐10 (A), IL‐4 (B), IL‐6 (C), TNF‐α (D). Results are expressed in pg/μL. Data are shown as mean ± SD, *n* = 30. **p < 0.01.

The IBS patients showed significantly lower IL‐10 serum levels than controls, 178.04 ± 143.65 vs. 383.34 ± 163.82 pg/μL (*p* < 0.01) (Figure 2A) and lower IL‐4: 286.86 ± 39.80 vs. 927.75 ± 298.08 pg/μL (*p* < 0.01) (Figure 2B). Also, there were higher levels of proinflammatory interleukins in patients with IBS vs. controls, IL‐6: 945.82 ± 271.74 vs. 286.88 ± 39.77 pg/μL (*p* < 0.01) (Figure 2C) and TNF‐α: 927.75 ± 298.08 vs. 286.85 ± 39.80 pg/μL (*p* < 0.01) (Figure 2D).

**FIGURE 3:**
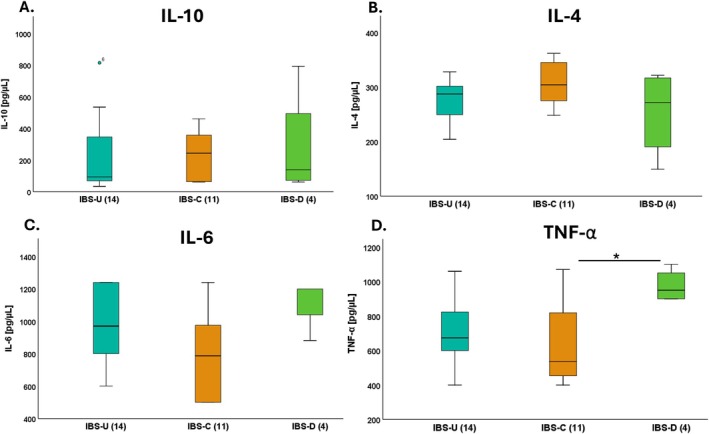
Mean serum levels of inflammatory and anti‐inflammatory interleukins in different bowel habit subtypes of IBS. IBS‐U, unclassified predominance; IBS‐C, constipation predominance; IBS‐D, diarrhea predominance. Graphs show the interleukin levels of IL‐10 (A), IL‐4 (B), IL‐6 (C), and TNF‐α (D). Results are expressed in pg/μL. Data are shown as mean ± SD, *n* = 29. *p < 0.05.

Figure 3 shows the serum levels of anti‐inflammatory and pro‐inflammatory interleukins according to the IBS subtypes (IBS‐U, IBS‐C, and IBS‐D). We only found higher levels of TNF‐α between IBS‐D: 975 ± 95.74 and IBS‐C: 645.36 ± 243.96 (*p* < 0.05) (Figure 3D) and there were no differences in IL‐10, IL‐4, and IL‐6 between the IBS subtypes (Figure 3A–C). In addition, there were no differences in the interleukins according to the IBS severity (Table S2).

### 3.4 Correlation Between OS and Interleukins

**FIGURE 4:**
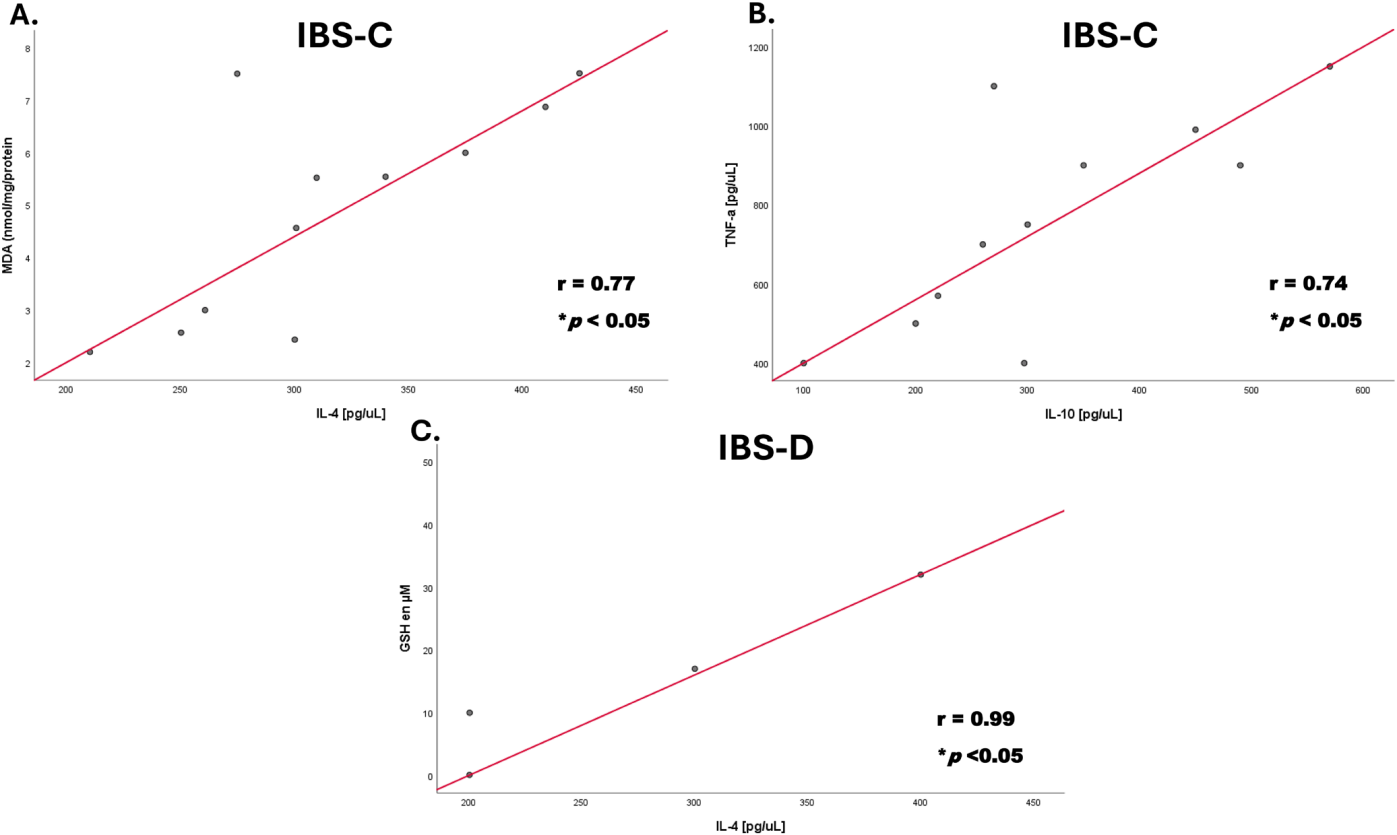
Correlation in different bowel habit subtypes of IBS. (A) Positive correlation between MDA and IL‐4 in IBS‐C. (B) Positive correlation between TNF‐α and IL‐10 in IBS‐C. *n* = 11. (C) Positive correlation between reduced glutathione (GSH) and IL‐4, *n* = 4. Pearson correlation *p < 0.05.

There were no significant correlations between each interleukin (IL‐10, IL‐4, IL‐6, TNF‐α) and the OS biomarkers (MDA, GSH, GSSG) in patients with IBS and controls (data not shown). However, according to the IBS subtypes, in IBS‐C, MDA positively correlated with IL‐4: *r* = 0.74 (*p* < 0.05) (Figure 4A) and TNF‐α with IL‐10: *r* = 0.75 (*p* < 0.05) (Figure 4B). In contrast, in IBS‐D, there was a positive correlation between GSH and IL‐4: *r* = 0.95 (*p* < 0.05) (Figure 4C).

### 3.5 Expression Proteins

**FIGURE 5:**
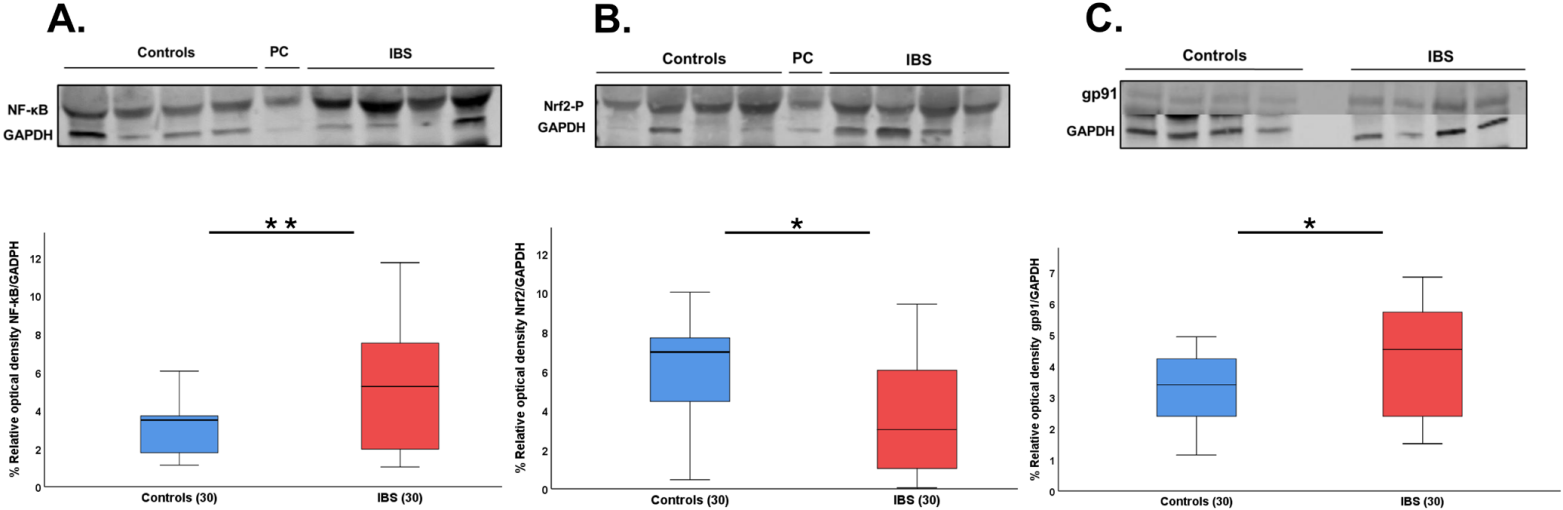
Expression of the proteins in peripheral blood of patients with IBS and controls. The figure shows representative blots performed for (A) NF‐kB (p65 subunit), (B) Nrf2, and (C) gp91. Densitometric analysis was normalized with GAPDH (as loading control). Results represent the mean ± SD of three independent experiments, *n* = 30. *p < 0.05; **p < 0.001. PC: Positive control.

In patients with IBS vs. controls, there was greater expression of NF‐κB: 5.33 ± 3.39 vs. 3.08 ± 1.19 (*p* < 0.01) (Figure 5A) and lower expression of Nrf2: 3.87 ± 2.99 vs. 7.56 ± 2.59 (*p* < 0.05) (Figure 5B). In addition, the expression of gp91^phox^ subunit of NADPH oxidase was higher in IBS vs. controls: 4.28 ± 1.81 vs. 3.29 ± 1.03 (*p* < 0.05) (Figure 5C). Finally, there were no differences according to the IBS subtypes (data not shown).

## 4 Discussion

The lack of an orchestrator in the pathophysiology of IBS is probably the most meaningful factor in the research of DGBI. In recent years, the emerging theory of activation of the immune system and consequently low‐grade inflammation as a key factor in IBS has become quite intrusive. An imbalance between anti‐ and pro‐inflammatory interleukins has been shown by different groups, and this may contribute to the low‐grade inflammation in IBS [33]. On the other hand, several interleukins can activate the NF‐κB which plays a central role in the inflammatory and immune response in humans, and chronic inflammation can sustain OS, while Nrf2 plays a crucial role in protecting cells from OS. Therefore, the interaction between Nrf2 and NF‐κB is essential to maintain the physiological homeostasis of the redox state and regulate the response to stress and inflammation [24]. In addition, a proper balance of NADPH oxidase activity is also essential for the maintenance of redox homeostasis [28, 29]. However, the proper balance between NF‐κB, Nrf2, and NADPH oxidase activity, or their relationship with OS, and anti‐and pro‐inflammatory interleukins has not been previously investigated in IBS. Therefore, in the current study, we investigated biomarkers of OS, serum levels of the anti‐inflammatory interleukins IL‐10 and IL‐4, and the pro‐inflammatory TNF‐α and IL‐6, as well as the mediators of the redox homeostasis NF‐κB and Nrf2, as well as the catalytic subunit gp91^phox^ of NADPH oxidase, in Rome III IBS patients and controls. We found higher expression of OS biomarkers in IBS vs. controls, including GSSG and MDA, and a lower concentration of the antioxidant GSH. In addition, there were significantly lower levels of IL‐10 and IL‐4, and higher IL‐6 and TNF‐α in IBS as previously reported. Also, TNF‐α was the only interleukin that was different according to the IBS subtype, being higher in IBS‐D. Finally, patients with IBS showed greater expression of NF‐κB and lower expression of Nrf2 compared to controls and higher expression of gp91^phox^. These findings support the concept of an imbalance in the redox state and an alteration in the regulation of OS that influences the low‐grade inflammation in IBS.

The imbalance of anti and pro‐inflammatory interleukins in IBS has been widely reported [34, 35]. This imbalance seems to be genetically determined, especially in IBS‐D for IL‐10 and TNF‐α [36] which agrees with our findings of higher TNF‐α levels in this IBS subtype. On the other hand, the elevated pro‐inflammatory IL‐6 and TNF‐α that we observed in IBS compared to controls can be related to their release in response to various stimuli, such as stress, diet, altered gut microbiota, or dysfunction of the enteric nervous system. IL‐6 is a multifunctional cytokine that is produced in response to inflammation, can be secreted by mast cells, and has been shown to induce direct activation of submucosal secretomotor neurons, thereby modulating intestinal function [37].

In addition, evidence suggests that OS may contribute to inflammation; therefore, OS may be an important factor in the pathophysiology of IBS [38, 39, 40]. OS can disrupt the integrity of the intestinal barrier, leading to increased permeability (leaky gut) [29]. A compromised intestinal barrier allows harmful substances, such as bacteria, toxins, and undigested food particles, to enter the bloodstream and activate immune responses, triggering inflammation and gastrointestinal symptoms characteristic of IBS [41]. Also, OS may sensitize visceral afferent nerves in the gut, leading to an increased perception of pain and discomfort in individuals with IBS [42]. The previous studies that analyzed OS in IBS found significantly higher levels of the lipid peroxidation product MDA, lower levels of antioxidant enzymes such as glutathione peroxidase, and total antioxidant capacity [38, 39]. These studies are in agreement to our results of increased markers of OS in IBS (MDA, GSSG) and lower concentrations of antioxidants (GSH), compared to controls. Notwithstanding, to the best of our knowledge, this is the first study that investigates the correlation between OS and interleukins in IBS and its different subtypes. This is important as OS can promote inflammation in the gastrointestinal tract, leading to the release of pro‐inflammatory cytokines and chemokines [43]. In addition, the results herein suggest differences in the underlying pathophysiological mechanisms according to the IBS subtype. Our finding of a positive correlation between GSH and IL‐4 levels in IBS‐D, in contrast to a positive correlation of MDA and IL‐4, and between TNF‐ α and IL‐10 in IBS‐C, suggests that in IBS‐D, the mechanism to counteract the low‐grade inflammatory is possibly given by an antioxidant effect, whereas in IBS‐C it is mainly given by interleukins. These differences may potentially provide therapeutic biomarkers for these subgroups. Interestingly, in the most common IBS subtype among our patients, IBS‐U, we did not observe any correlations between OS and interleukins, suggesting that immune activation is not a fundamental pathophysiological mechanism related to this group, but further studies are required to clarify these findings.

On the other hand, the transcription factor NF‐κB regulates multiple aspects of innate and adaptive immune functions and serves as a fundamental mediator of the inflammatory response. Its activation leads to the expression of pro‐inflammatory genes such as cytokines and chemokines, which recruit and activate immune system cells at the site of inflammation [44]. Our analysis showed that NF‐κB is mainly expressed in IBS patients compared to controls. This is consistent with a previous report on a rat model for IBS that investigated the NF‐κB signaling pathways in the colon. In that study, the administration of oridonin, a compound with anti‐inflammatory effects, inhibited the phosphorylation and expression of NF‐κB [45]. However, the reach of NF‐κB extends transcriptional regulation beyond the limits of the immune response, acting broadly to influence gene expression events that affect cell survival, differentiation, and proliferation [46].

With regards to the transcription factor Nrf2, it plays a critical role in defending against OS and preserving cellular homeostasis by inducing the expression of antioxidant enzymes and suppressing inflammation. Nrf2 is relevant for the maintenance and proper functionality of the gastrointestinal tract [47]. It has also shown promise in the prevention of intestinal fibrosis and colorectal cancer in inflammatory bowel disease [48]. In another study on intestinal porcine epithelial cells, quercetin, a flavonoid with antioxidant and anti‐inflammatory properties, abrogated a diquat‐induced oxidative stress in the enterocytes. It was determined that the protective effect of quercetin was associated with elevated protein abundance of Nrf2 and increased intracellular GSH content [49] The above studies support our results and indicate that the transcription factor NF‐κB and Nrf2 may act synergistically to stimulate the immune response.

The deregulation of the redox balance compromises antimicrobial defenses and alters innate and adaptive immune responses. NADPH oxidase are also an important producer of ROS, which are involved in the maintenance of a healthy epithelial barrier in the gut and have antimicrobial activity [27]. NADPH oxidase‐induced ROS signals are associated with immune cell activation and inflammation in the gastrointestinal tract [50]. There are two mechanisms for the above: first, the inflammatory stimulation through bacterial lipopolysaccharides induces NADPH oxidase expression in macrophages and ROS signaling, further increasing inflammatory signals and immune response in macrophages; secondly, inflammation in the colon upregulates NADPH oxidase expression and induces ROS production and signaling, also increasing the immune response, while NADPH oxidase inhibition abrogates ROS‐mediated NF‐κB signaling and the immune response in the colon [51]. The above may explain our observation of a higher expression of the catalytic subunit gp91^phox^ of NADPH, suggesting that the immune system cells may be deregulated and generate an OS environment.

Elucidating the above‐mentioned mechanisms is crucial for advancing the diagnosis and management of IBS, given that many signaling pathways have redundancies or compensatory mechanisms that hinder the success of targeted therapies. However, the current study has some limitations. First, the absence of relevant demographic and clinical data from patients, such as BMI and dietary habits, may be important as these factors could have a significant impact on the levels of OS and inflammation markers. The lack of this information could influence the interpretation of the results and may affect the generalization of the findings. Notwithstanding, in a previous study also comparing OS between Rome III‐IBS patients and controls, we did not find any differences according to BMI [52]. A second limitation is the small sample size within each IBS subtype to draw conclusions about OS between these subgroups, especially considering the exclusion of IBS‐M because it was almost absent among our patients. Our sample size calculation was powered to detect differences between IBS and controls, but not between IBS subtypes. However, we thought that conducting comparisons between the IBS subtypes was important as it allows the identification of specific differences to be addressed in future research studies. An additional issue but not a limitation of this research is the use of the Rome III criteria for the diagnosis of IBS instead of the more recent Rome IV criteria. Subjects' recruitment began before the official Rome IV questionnaire had been translated into Spanish‐Mexico, and therefore, the Rome III criteria were used. Nonetheless, it is important to highlight that the Rome criteria have been developed to standardize patients that are included in a research protocol, and using previous iterations of Rome criteria does not invalidate a study. Although the majority of our Rome III patients were moderate to severe according to the IBS‐SSS, if anything, using Rome IV criteria may have identified even more severe IBS patients, as the Rome IV criteria for IBS are stricter. In fact, using Rome IV criteria compared to Rome III criteria decreases the prevalence of IBS by half [53]. Thus, this methodological choice does not disprove our findings, although it might affect the comparability of the results with studies utilizing more recent Rome iterations. A final limitation of this study is that the measurement of cytokines and inflammation markers was performed on systemic samples rather than directly in intestinal tissue. Although this may not fully capture the local inflammatory events in the bowel, systemic levels of these markers can provide an indirect view of the underlying inflammatory processes in IBS [33, 54]. However, our findings of lower levels of anti‐inflammatory interleukins and higher pro‐inflammatory interleukins in IBS are consistent with previous research studies in IBS and are supported by a robust methodology, which reinforces their validity and relevance. Most existing studies on IBS focus on investigating specific aspects of its pathophysiology in isolation, such as gut dysbiosis [55, 56], OS [38, 40], or individual inflammatory markers [57, 58, 59, 60]. However, the multifactorial nature of IBS suggests that an integrated view of multiple biological pathways is essential to understanding the underlying mechanisms. In this study, we have simultaneously analyzed a variety of key markers related to inflammation, covering both OS and the immune response. This approach not only allows for a more complete characterization of the inflammatory/immunological process in IBS but also provides new insights into the interactions between these pathways. Thus, we believe that our work represents a significant contribution to literature as it addresses the need for integrative approaches that better reflect the complexity of the underlying mechanisms of IBS. On the other hand, advances in IBS research may serve as a model for other multifactorial conditions, highlighting the importance of integrated approaches in the study of complex diseases.

## 5 Conclusions

The results obtained in this research study suggest the participation of the enzyme NADPH oxidase as part of the activation of the immune system leading to an increased production of ROS generating OS in IBS. Further, OS can activate signaling pathways that lead to the activation and translocation of NF‐κB to the nucleus inducing the transcription of proinflammatory genes, such as proinflammatory interleukins. Consequently, a dysfunction in regulation between the transcription factors NF‐κB and Nrf2 may lead to increased susceptibility to inflammation and oxidative damage. This cascade of events connects OS to inflammation and highlights the interconnection between the immune system. It is important to highlight that these mechanisms do not act in isolation but are interrelated and can jointly contribute to the development and clinical manifestation of IBS symptoms.

## Author Contributions

Application of questionnaires, sample collection and processing, standardization of techniques, conducting experiments, analysis of results, and article writing: A. S. Morales Guzmán. Concept and design of the study, determination of oxidant stress biomarkers, critical writing, and revision of the manuscript: A. Alarcón‐Aguilar. Study concept and design, evaluation of protein expression (NF‐κB, Nrf2, and gp91^phox^), critical writing and manuscript review: A. Luna‐López. Biostatistical analysis and data interpretation: A. D. Santana‐Vargas. Collection of patients and controls: M. Motola‐Kuba. Standardization of techniques, experimental work: Librado‐Osorio Raúl. Determination of pro‐inflammatory and anti‐inflammatory interleukins: García‐Álvarez Jorge Antonio. Study concept and design, data acquisition, data analysis, and interpretation, critical writing, and manuscript writing: M. J. Schmulson. All authors reviewed and approved the final manuscript.

## Conflicts of Interest

The authors declare no conflicts of interest.

## Supporting information


**Table S1.** Oxidative stress markers according to IBS severity. The table shows the values of OS markers (reduced glutathione [GSH], oxidized glutathione [GSSG], GSH/GSSG ratio, malondialdehyde [MDA], and protein carbonyls [PC]) in IBS patients stratified in mild, moderate, and severe groups. Data are shown in mean ± SD, *n* = 30. *p < 0.05.

**Table S2.** Interleukins levels according to IBS severity. The table shows the interleukins IL‐10, IL‐4, IL‐6, and tumor necrosis factor‐alpha (TNF‐α) in IBS patients stratified in mild, moderate, and severe groups. Data are shown as mean ± SD, *n* = 30. *p < 0.05.

## Data Availability

The data that support the findings of this study are available on request from the corresponding author. The data are not publicly available due to privacy or ethical restrictions.
