# Supplementary material for: Abnormal Homeostasis in the Redox State and Related Signaling Pathways, in Irritable Bowel Syndrome
Source: Neurogastroenterol Motil. 2025 Jun 22;37(12):e70097. doi: 10.1111/nmo.70097 (PMC12623273; doi:10.1111/nmo.70097)
Supplement: Supplementary file 1 — Table S1. Oxidative stress markers according to IBS severity. The table shows the values of OS markers (reduced glutathione [GSH], oxidized glutathione [GSSG], GSH/GSSG ratio, malondialdehyde [MDA], and protein carbonyls [PC]) in IBS patients stratified in mild, moderate, and severe groups. Data are shown in mean ± SD, n = 30. *p < 0.05. [file NMO-37-e70097-s001.docx]

**Supplemental Table 1.**

| **IBS-SSS** | **GSH/GSSG ratio** | **GSH (μM)** | **GSSG (μM)** | **MDA (nmol/mg/protein)** | **PC (nmol/mg/protein)** |
| --- | --- | --- | --- | --- | --- |
| **Mild**  **(4)** | 2.2±1.2 | 26.2±16.5 | 58.5±18.1 | 3.9±.08 | 45.5±6.9 |
| **Moderate (12)** | 2.1±0.8 | 25±10 | 58.7±17.2 | 4.5±1.9 | 40.7±12 |
| **Severe**  **(14)** | 2.6±1.2 | 27.1±13.8 | 55.4±18.7 | 4.4±1.8 | 44.3±12.2 |
| **p** | 0.6 | 0.9 | 0.9 | 0.7 | 0.7 |
