# Supplementary material for: Abnormal Homeostasis in the Redox State and Related Signaling Pathways, in Irritable Bowel Syndrome
Source: Neurogastroenterol Motil. 2025 Jun 22;37(12):e70097. doi: 10.1111/nmo.70097 (PMC12623273; doi:10.1111/nmo.70097)
Supplement: Supplementary file 2 — Table S2. Interleukins levels according to IBS severity. The table shows the interleukins IL‐10, IL‐4, IL‐6, and tumor necrosis factor‐alpha (TNF‐α) in IBS patients stratified in mild, moderate, and severe groups. Data are shown as mean ± SD, n = 30. *p < 0.05. [file NMO-37-e70097-s002.docx]

**Supplemental Table 2.**

|  | **IL-10 (pg/µL)** | **IL-4 (pg/µL)** | **IL-6 (pg/µL)** | **TNF-α (pg/µL)** |
| --- | --- | --- | --- | --- |
| **Mild**  **(4)** | 326.2±57.8 | 282.6±35.5 | 525.5±49.9 | 437.3±32.5 |
| **Moderate**  **(12)** | 111.9±67.6 | 286.2±37.6 | 1051.4±252.7 | 1047.4±262.2 |
| **Severe**  **(14)** | 192.3±174.3 | 288.5±45.0 | 975.3±210.9 | 965.2±225.8 |
| **p** | 0.1 | 0.9 | 0.7 | 0.7 |
